# Supplementary figures and images for: Housing starts and the associated wood products carbon storage by county by Shared Socioeconomic Pathway in the United States
Source: PLoS One. 2022 Aug 11;17(8):e0270025. doi: 10.1371/journal.pone.0270025 (PMC9371325; doi:10.1371/journal.pone.0270025)

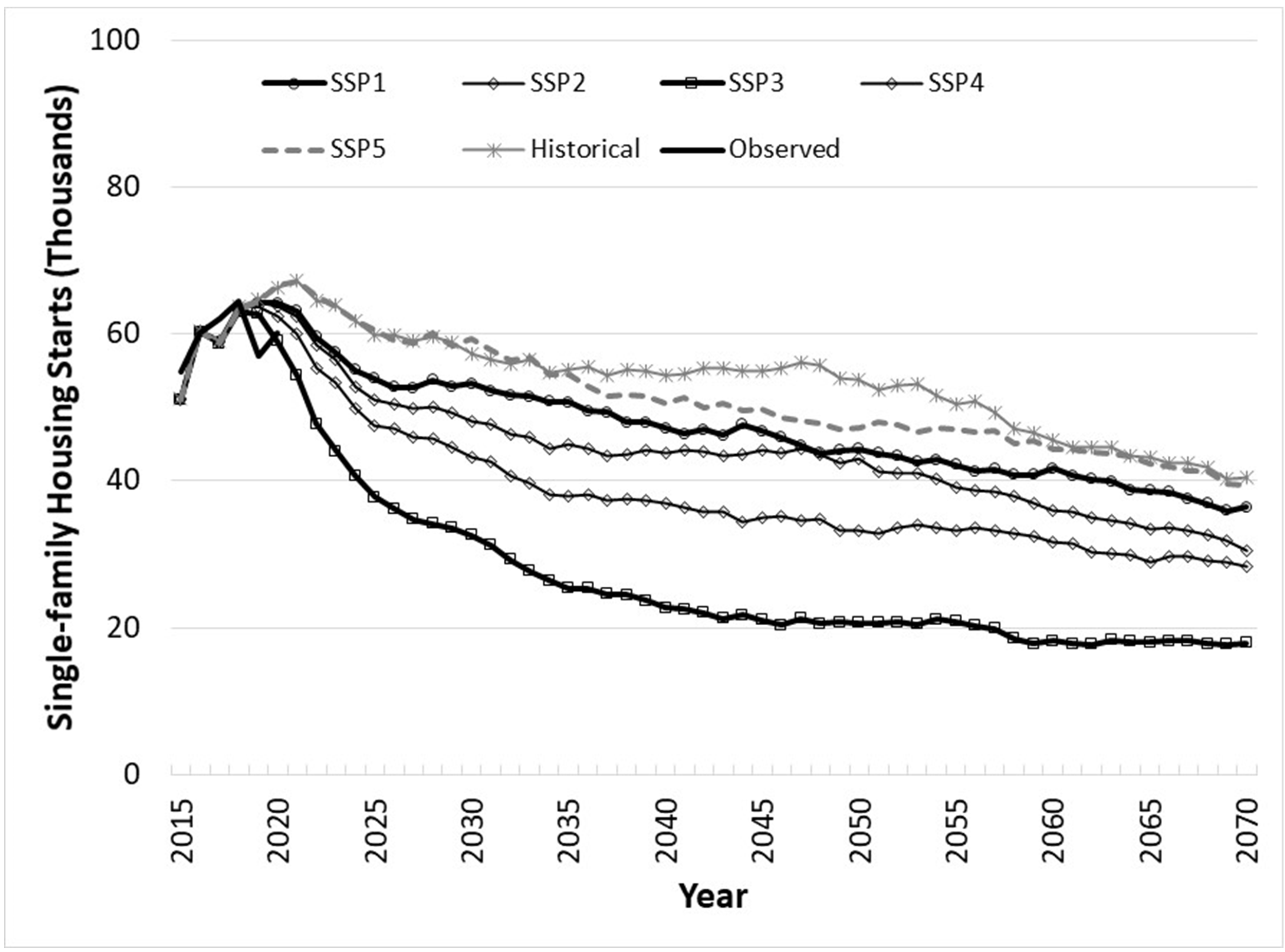

Supplement: S1 Fig — (TIF) [file pone.0270025.s001.TIF]

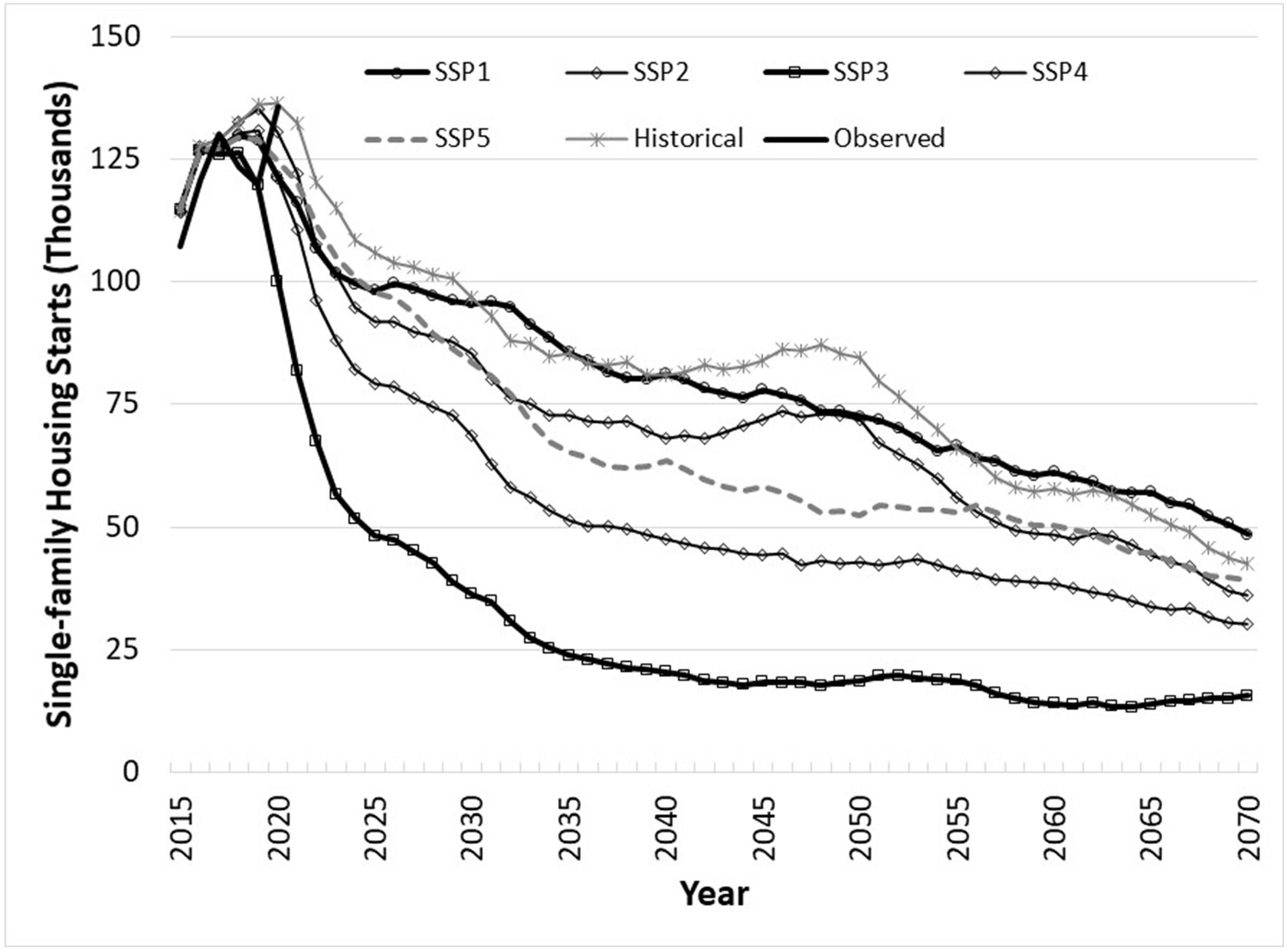

Supplement: S2 Fig — (TIF) [file pone.0270025.s002.TIF]

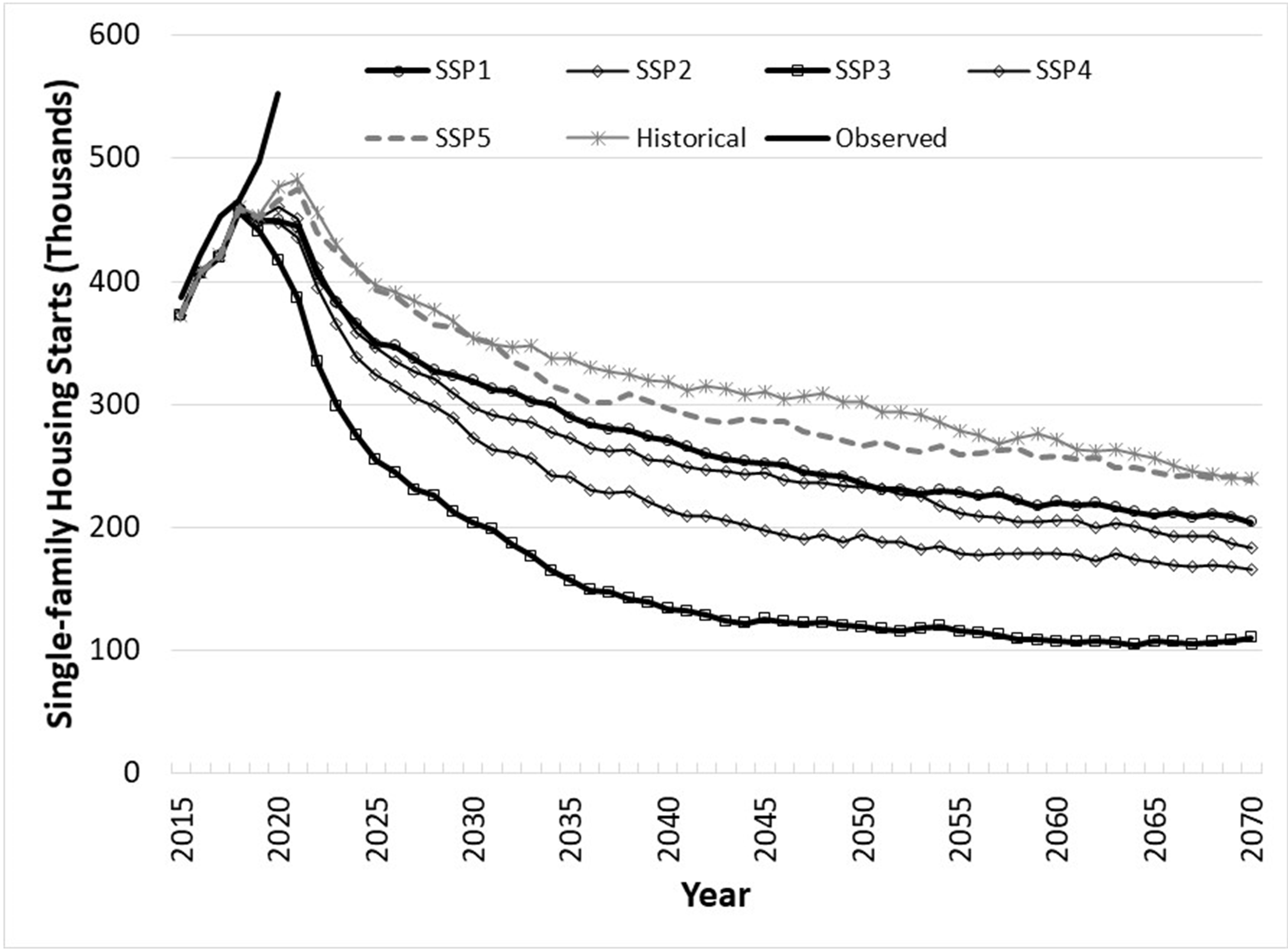

Supplement: S3 Fig — (TIF) [file pone.0270025.s003.TIF]

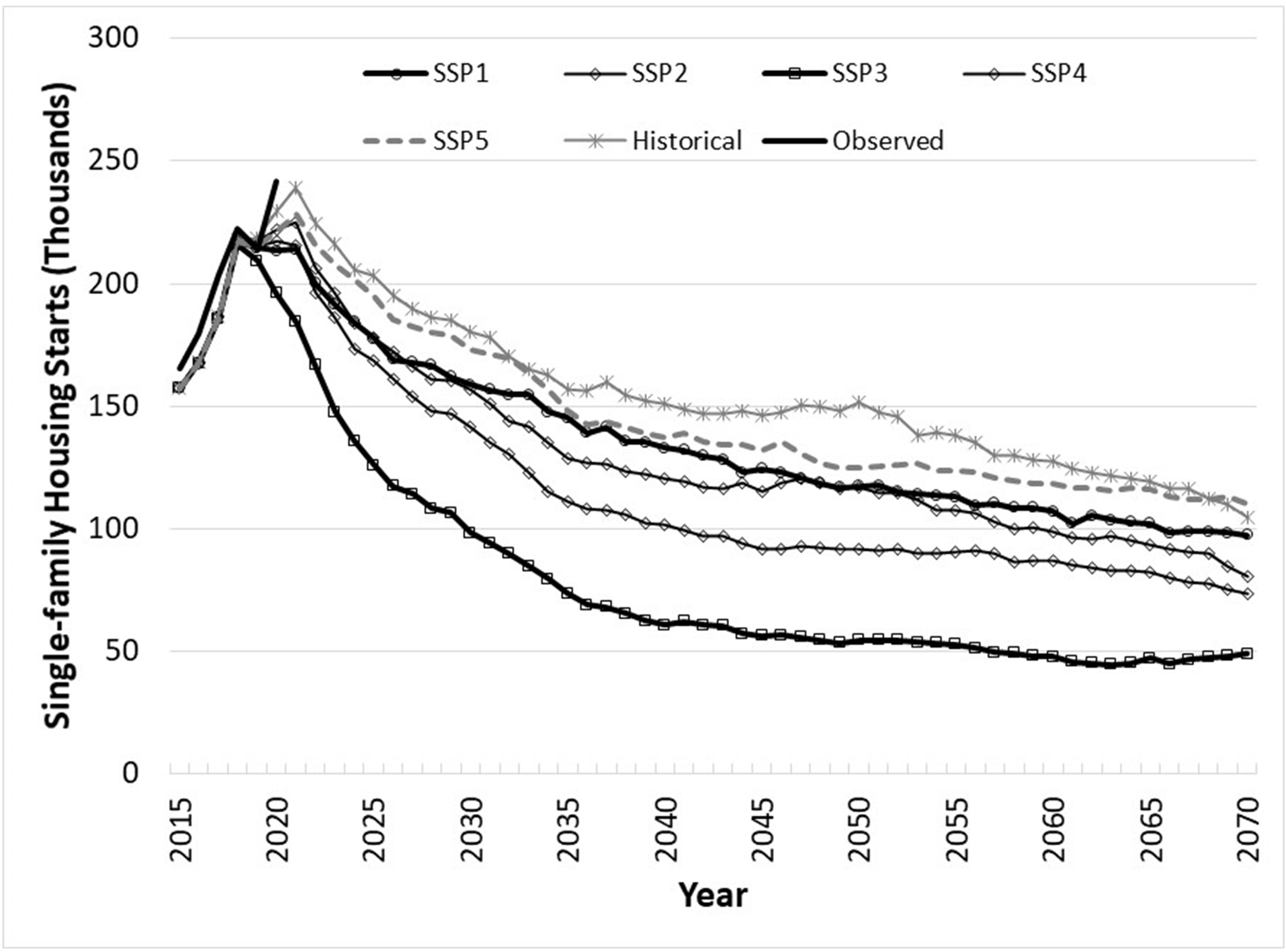

Supplement: S4 Fig — (TIF) [file pone.0270025.s004.TIF]

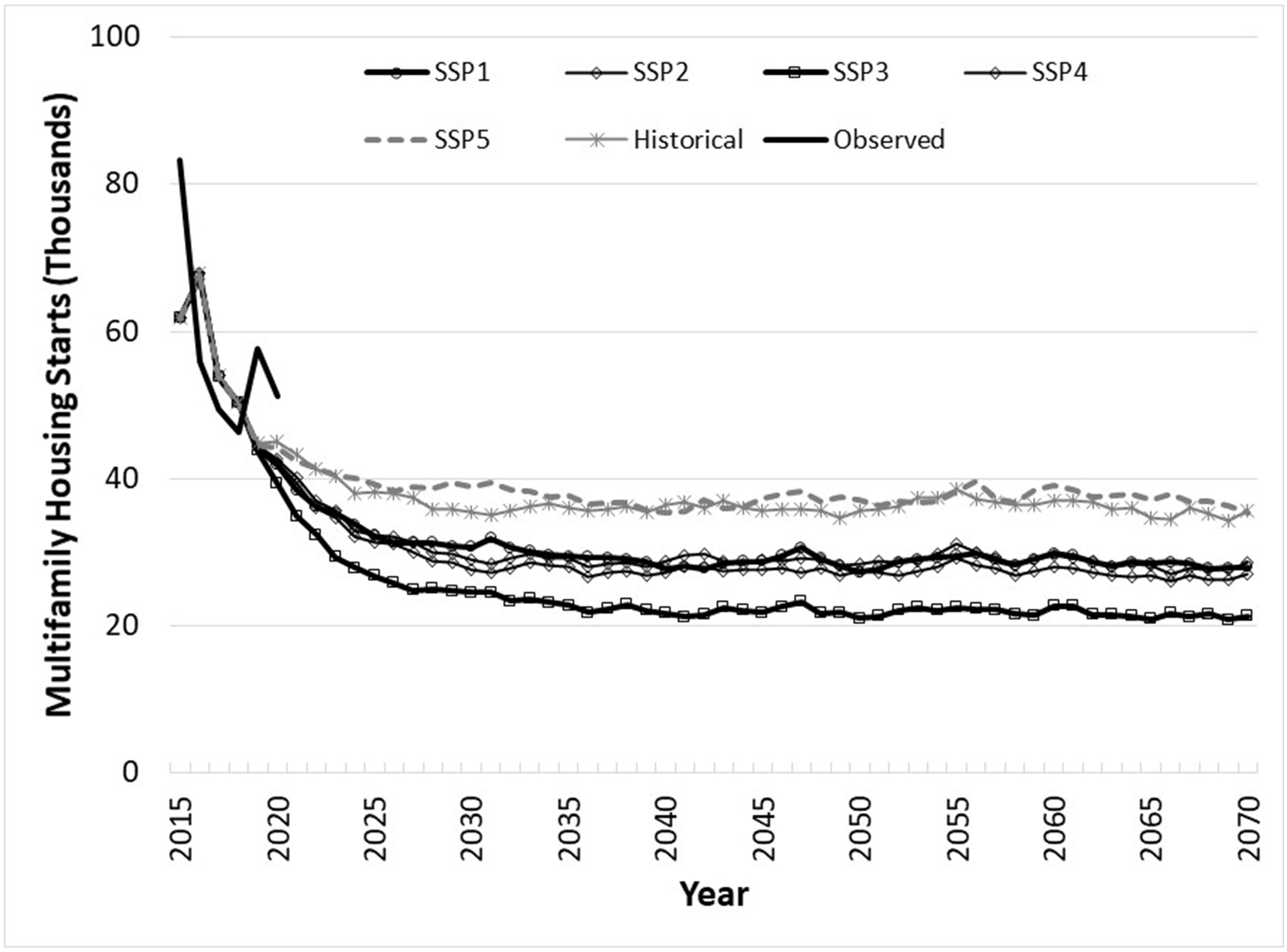

Supplement: S5 Fig — (TIF) [file pone.0270025.s005.TIF]

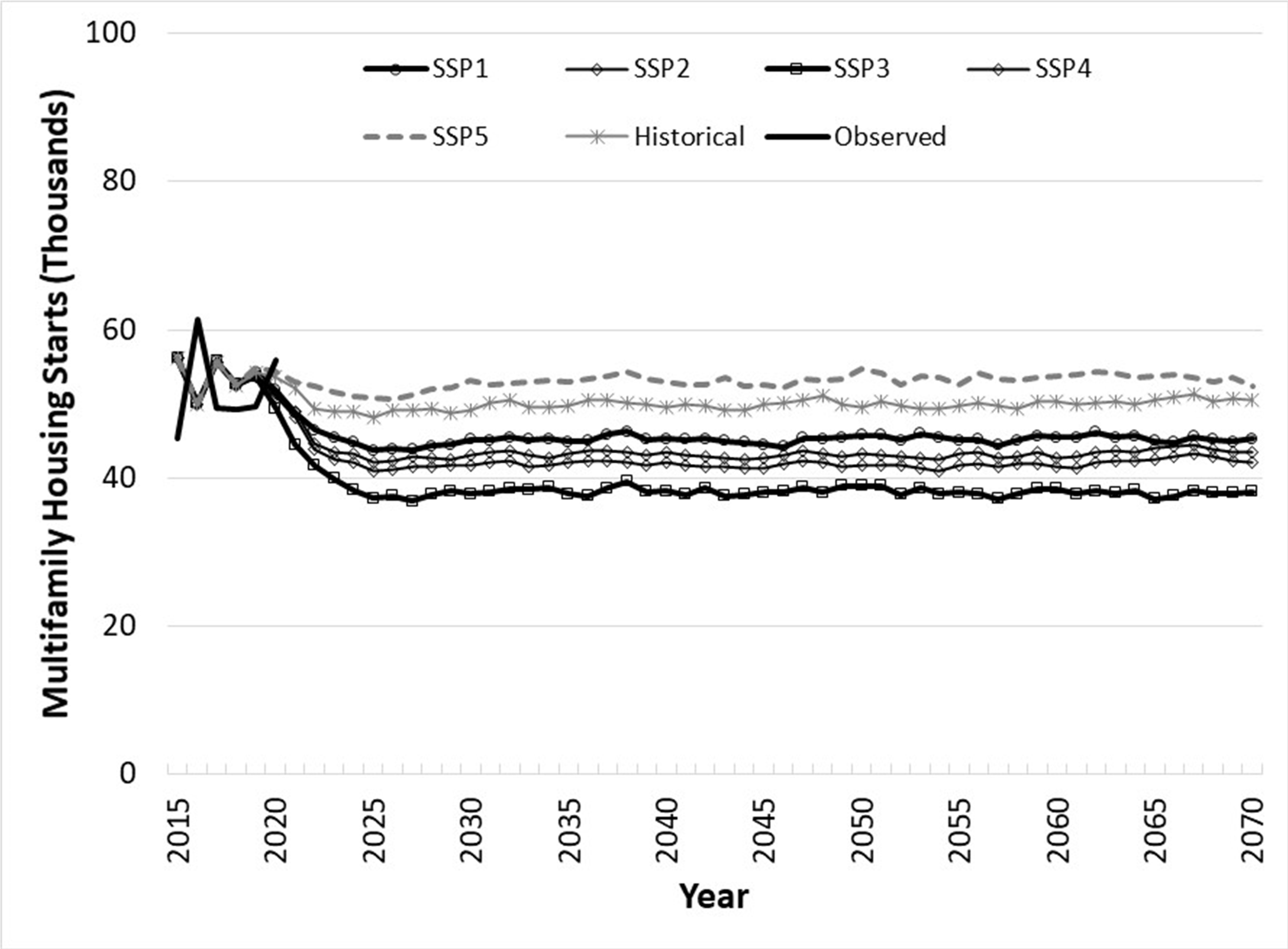

Supplement: S6 Fig — (TIF) [file pone.0270025.s006.TIF]

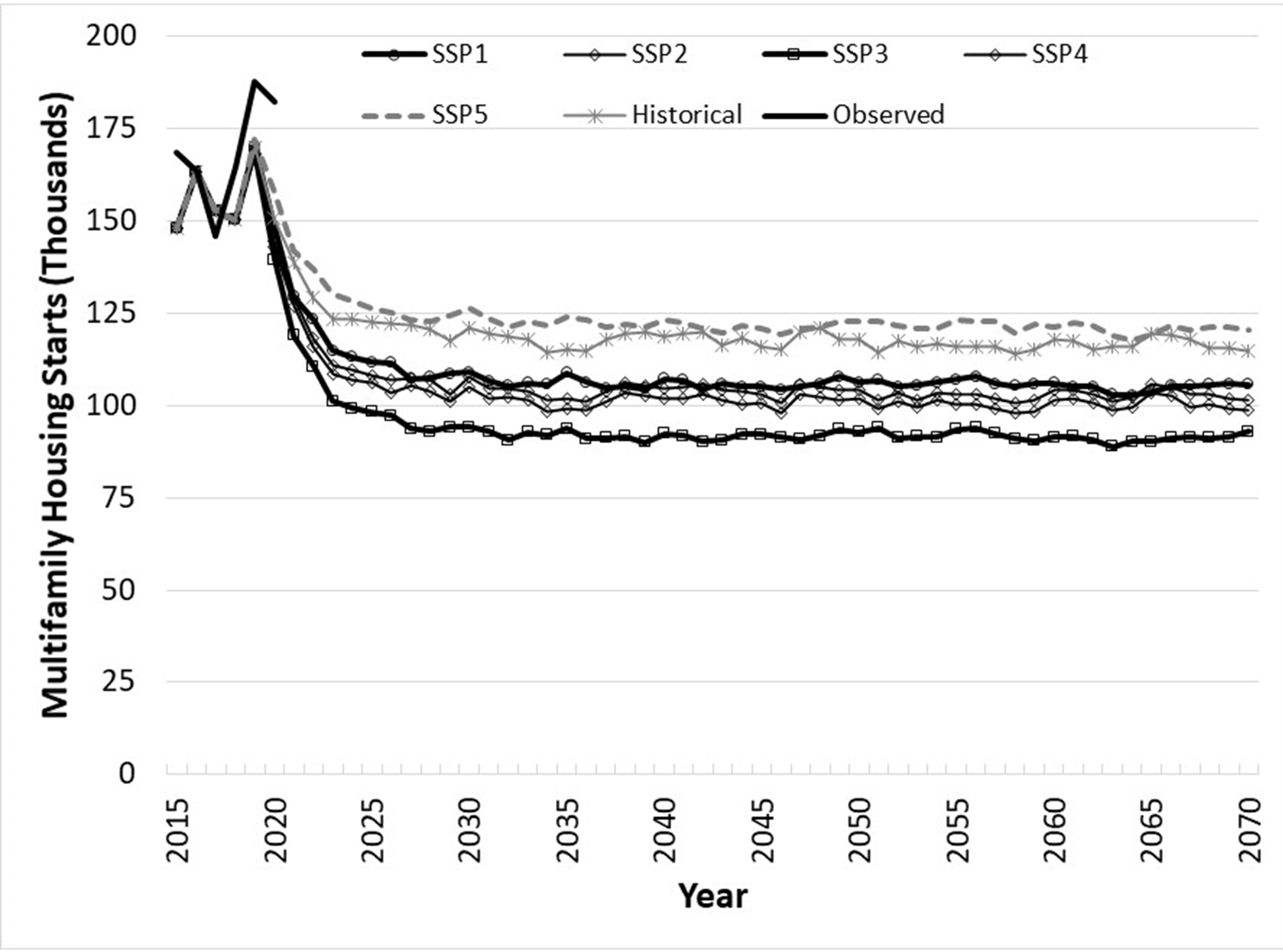

Supplement: S7 Fig — (TIF) [file pone.0270025.s007.TIF]

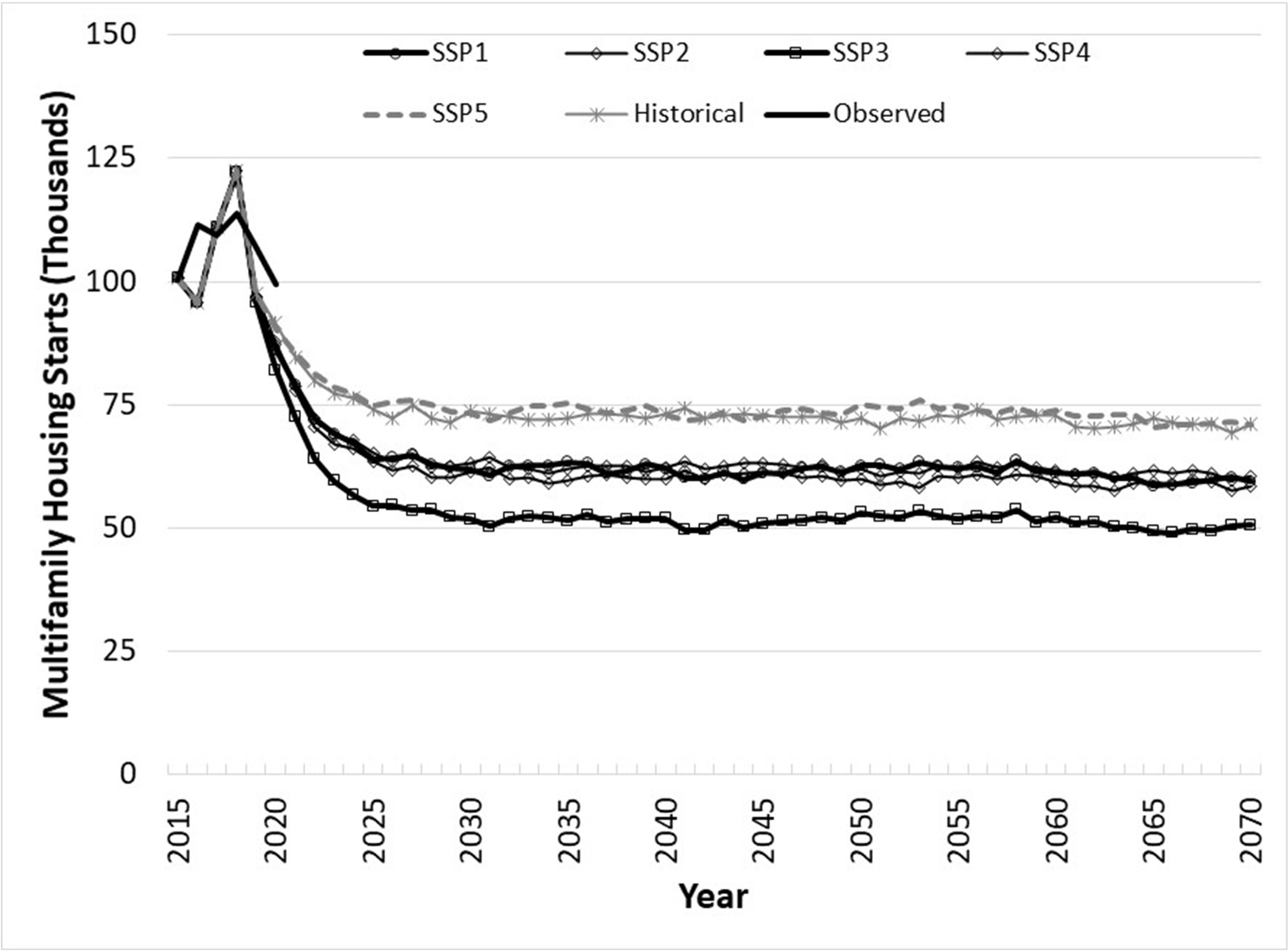

Supplement: S8 Fig — (TIF) [file pone.0270025.s008.TIF]
