# Supplementary material for: Housing starts and the associated wood products carbon storage by county by Shared Socioeconomic Pathway in the United States
Source: PLoS One. 2022 Aug 11;17(8):e0270025. doi: 10.1371/journal.pone.0270025 (PMC9371325; doi:10.1371/journal.pone.0270025)
Supplement: S1 Table — (DOCX) [file pone.0270025.s009.docx]

S1 Table. Northeast U.S. Census Region quarterly total (single-family + multifamily) housing starts, Poisson pseudo-maximum likelihood equation estimates.

|  | Coefficient | Standard Error | t-value | p-value |
| --- | --- | --- | --- | --- |
| Northeast Total Starts(t-1) | 0.015 | 0.002 | 7.94 | 0.00 |
| Q1 | -0.24 | 0.08 | -2.78 | 0.01 |
| Q2 | 0.39 | 0.05 | 7.19 | 0.00 |
| Q3 | 0.14 | 0.04 | 3.54 | 0.00 |
| D(Ln(US real GDP)) | 7.46 | 3.10 | 2.40 | 0.02 |
| D(Mortgage Delinquency Rate) | -0.13 | 0.07 | -1.88 | 0.06 |
| D(Mortgage Rate(t-1)) | -0.080 | 0.038 | -2.12 | 0.03 |
| D(U.S. Total Population) | 43.86 | 31.90 | 1.37 | 0.17 |
| Northeast Total Starts(t-4) | 0.0060 | 0.0017 | 3.42 | 0.00 |
| Constant | 2.52 | 0.10 | 24.52 | 0.00 |
| Number of Observations | 122 |  |  |  |
| Wald χ^2^ (9) | 774.64 |  |  |  |
| Prob > χ^2^ | 0.00 |  |  |  |
| Pseudo R^2^ | 0.48 |  |  |  |
