# Supplementary material for: Housing starts and the associated wood products carbon storage by county by Shared Socioeconomic Pathway in the United States
Source: PLoS One. 2022 Aug 11;17(8):e0270025. doi: 10.1371/journal.pone.0270025 (PMC9371325; doi:10.1371/journal.pone.0270025)
Supplement: S2 Table — (DOCX) [file pone.0270025.s010.docx]

S2 Table. Midwest U.S. Census Region quarterly total (single-family + multifamily) housing starts, Poisson pseudo-maximum likelihood equation estimates.

|  | Coefficient | Standard Error | t-value | p-value |
| --- | --- | --- | --- | --- |
| Midwest Total Starts(t-1) | 0.012 | 0.001 | 12.52 | 0.00 |
| Q1 | -0.19 | 0.05 | -3.70 | 0.00 |
| Q2 | 0.57 | 0.06 | 9.47 | 0.00 |
| Q3 | 0.12 | 0.02 | 4.90 | 0.00 |
| D(Ln(US real GDP)) | 7.83 | 1.71 | 4.59 | 0.00 |
| D(Mortgage Delinquency Rate) | -0.11 | 0.04 | -2.88 | 0.00 |
| D(Mortgage Rate(t-1)) | -0.10 | 0.02 | -4.45 | 0.00 |
| D(U.S. Total Population) | 90.73 | 20.33 | 4.46 | 0.00 |
| Midwest Total Starts(t-4) | 0.0046 | 0.0010 | 4.62 | 0.00 |
| Constant | 2.60 | 0.08 | 31.10 | 0.00 |
| Number of Observations | 122 |  |  |  |
| Wald χ^2^ (9) | 1487.81 |  |  |  |
| Prob > χ^2^ | 0.00 |  |  |  |
| Pseudo R^2^ | 0.62 |  |  |  |
