# Supplementary material for: Housing starts and the associated wood products carbon storage by county by Shared Socioeconomic Pathway in the United States
Source: PLoS One. 2022 Aug 11;17(8):e0270025. doi: 10.1371/journal.pone.0270025 (PMC9371325; doi:10.1371/journal.pone.0270025)
Supplement: S3 Table — (DOCX) [file pone.0270025.s011.docx]

S3 Table. South U.S. Census Region quarterly total (single-family + multifamily) housing starts, Poisson pseudo-maximum likelihood equation estimates.

|  | Coefficient | Standard Error | t-value | p-value |
| --- | --- | --- | --- | --- |
| South Total Starts(t-1) | 0.0062 | 0.0002 | 29.11 | 0.00 |
| Q1 | 0.082 | 0.036 | 2.30 | 0.02 |
| Q2 | 0.29 | 0.02 | 12.97 | 0.00 |
| Q3 | 0.075 | 0.021 | 3.67 | 0.00 |
| D(Ln(US real GDP)) | 5.75 | 1.49 | 3.85 | 0.00 |
| D(Mortgage Delinquency Rate) | -0.087 | 0.028 | -3.09 | 0.00 |
| D(Mortgage Rate(t-1)) | -0.060 | 0.020 | -3.04 | 0.00 |
| D(U.S. Total Population) | 75.05 | 17.88 | 4.20 | 0.00 |
| Constant | 3.68 | 0.07 | 50.85 | 0.00 |
| Number of Observations | 122 |  |  |  |
| Wald χ^2^ (8) | 1369.04 |  |  |  |
| Prob > χ^2^ | 0.00 |  |  |  |
| Pseudo R^2^ | 0.66 |  |  |  |
