# Supplementary material for: Housing starts and the associated wood products carbon storage by county by Shared Socioeconomic Pathway in the United States
Source: PLoS One. 2022 Aug 11;17(8):e0270025. doi: 10.1371/journal.pone.0270025 (PMC9371325; doi:10.1371/journal.pone.0270025)
Supplement: S4 Table — (DOCX) [file pone.0270025.s012.docx]

S4 Table. West U.S. Census Region quarterly total (single-family + multifamily) housing starts, Poisson pseudo-maximum likelihood equation estimates.

|  | Coefficient | Standard Error | t-value | p-value |
| --- | --- | --- | --- | --- |
| West Total Starts(t-1) | 0.012 | 0.001 | 23.91 | 0.00 |
| Q1 | 0.15 | 0.06 | 2.74 | 0.01 |
| Q2 | 0.44 | 0.03 | 15.08 | 0.00 |
| Q3 | 0.15 | 0.03 | 4.44 | 0.00 |
| D(Ln(US real GDP)) | 4.60 | 2.06 | 2.24 | 0.03 |
| D(Mortgage Delinquency Rate) | -0.061 | 0.039 | -1.56 | 0.12 |
| D(Mortgage Rate(t-1)) | -0.050 | 0.030 | -1.66 | 0.10 |
| D(U.S. Total Population) | 119.18 | 24.81 | 4.80 | 0.00 |
| Constant | 2.82 | 0.09 | 30.44 | 0.00 |
| Number of Observations | 122 |  |  |  |
| Wald χ^2^ (8) | 905.70 |  |  |  |
| Prob > χ^2^ | 0.00 |  |  |  |
| Pseudo R^2^ | 0.61 |  |  |  |
