# Supplementary material for: Housing starts and the associated wood products carbon storage by county by Shared Socioeconomic Pathway in the United States
Source: PLoS One. 2022 Aug 11;17(8):e0270025. doi: 10.1371/journal.pone.0270025 (PMC9371325; doi:10.1371/journal.pone.0270025)
Supplement: S5 Table — (DOCX) [file pone.0270025.s013.docx]

S5 Table. Northeast U.S. Census Region quarterly single-family housing starts, least squares equation estimates; dependent variable natural log.

|  | Coefficient | Standard Error | t-value | p-value |
| --- | --- | --- | --- | --- |
| Ln(Northeast Single-family Starts(t-1)) | 0.62 | 0.09 | 7.11 | 0.00 |
| Q1 | -0.33 | 0.06 | -5.31 | 0.00 |
| Q2 | 0.55 | 0.04 | 15.47 | 0.00 |
| Q3 | 0.31 | 0.05 | 5.77 | 0.00 |
| D(Ln(US real GDP Per Capita)) | 6.84 | 2.08 | 3.28 | 0.00 |
| D(Ln(Mortgage Delinquency Rate)) | -0.54 | 0.24 | -2.22 | 0.03 |
| D(Ln(Mortgage Rate(t-1))) | -0.28 | 0.18 | -1.55 | 0.12 |
| Ln(Northeast Single-family Starts(t-2)) | 0.33 | 0.08 | 3.97 | 0.00 |
| D(Ln(U.S. Total Population)) | 17.10 | 26.86 | 0.64 | 0.53 |
| Constant | -0.06 | 0.10 | -0.64 | 0.52 |
| Number of Observations | 121 |  |  |  |
| F(9,111) | 197.81 |  |  |  |
| Prob > F | 0.00 |  |  |  |
| R^2^ | 0.95 |  |  |  |
| Root MSE | 0.11 |  |  |  |
| Durbin’s H-Statistic | -0.35 |  |  |  |
