# Supplementary material for: Housing starts and the associated wood products carbon storage by county by Shared Socioeconomic Pathway in the United States
Source: PLoS One. 2022 Aug 11;17(8):e0270025. doi: 10.1371/journal.pone.0270025 (PMC9371325; doi:10.1371/journal.pone.0270025)
Supplement: S6 Table — (DOCX) [file pone.0270025.s014.docx]

S6 Table. Midwest U.S. Census Region quarterly single-family housing starts, least squares equation estimates; dependent variable natural log.

|  | Coefficient | Standard Error | t-value | p-value |
| --- | --- | --- | --- | --- |
| Ln(Midwest Single-family Starts(t-1)) | 0.93 | 0.03 | 31.54 | 0.00 |
| Q1 | -0.26 | 0.06 | -4.47 | 0.00 |
| Q2 | 0.87 | 0.03 | 30.55 | 0.00 |
| Q3 | 0.21 | 0.02 | 9.74 | 0.00 |
| D(Ln(US real GDP Per Capita)) | 6.01 | 2.04 | 2.94 | 0.00 |
| D(Ln(Mortgage Delinquency Rate)) | -0.80 | 0.22 | -3.63 | 0.00 |
| D(Ln(Mortgage Rate(t-1))) | -0.47 | 0.19 | -2.40 | 0.02 |
| D(Ln(U.S. Total Population)) | 54.59 | 26.47 | 2.06 | 0.04 |
| Constant | -0.10 | 0.10 | -1.04 | 0.30 |
| Number of Observations | 122 |  |  |  |
| F(8,113) | 328.89 |  |  |  |
| Prob > F | 0.00 |  |  |  |
| R^2^ | 0.96 |  |  |  |
| Root MSE | 0.10 |  |  |  |
| Durbin’s H-Statistic | -0.29 |  |  |  |
