# Supplementary material for: Housing starts and the associated wood products carbon storage by county by Shared Socioeconomic Pathway in the United States
Source: PLoS One. 2022 Aug 11;17(8):e0270025. doi: 10.1371/journal.pone.0270025 (PMC9371325; doi:10.1371/journal.pone.0270025)
Supplement: S7 Table — (DOCX) [file pone.0270025.s015.docx]

S7 Table. South U.S. Census Region quarterly single-family housing starts, least squares equation estimates; dependent variable natural log.

|  | Coefficient | Standard Error | t-value | p-value |
| --- | --- | --- | --- | --- |
| Ln(South Single-family Starts(t-1)) | 0.96 | 0.02 | 40.40 | 0.00 |
| Q1 | 0.16 | 0.03 | 5.60 | 0.00 |
| Q2 | 0.35 | 0.02 | 20.49 | 0.00 |
| Q3 | 0.12 | 0.02 | 6.09 | 0.00 |
| D(Ln(US real GDP Per Capita)) | 5.09 | 1.13 | 4.49 | 0.00 |
| D(Ln(Mortgage Delinquency Rate)) | -0.37 | 0.12 | -2.96 | 0.00 |
| D(Ln(Mortgage Rate(t-1))) | -0.43 | 0.14 | -3.20 | 0.00 |
| D(Ln(U.S. Total Population)) | 17.17 | 13.39 | 1.28 | 0.20 |
| Constant | -0.03 | 0.12 | -0.26 | 0.79 |
| Number of Observations | 122 |  |  |  |
| F(8,113) | 381.55 |  |  |  |
| Prob > F | 0.00 |  |  |  |
| R^2^ | 0.97 |  |  |  |
| Root MSE | 0.066 |  |  |  |
| Durbin’s H-Statistic | 0.10 |  |  |  |
