# Supplementary material for: Housing starts and the associated wood products carbon storage by county by Shared Socioeconomic Pathway in the United States
Source: PLoS One. 2022 Aug 11;17(8):e0270025. doi: 10.1371/journal.pone.0270025 (PMC9371325; doi:10.1371/journal.pone.0270025)
Supplement: S8 Table — (DOCX) [file pone.0270025.s016.docx]

S8 Table. West U.S. Census Region quarterly single-family housing starts, least squares equation estimates; dependent variable natural log.

|  | Coefficient | Standard Error | t-value | p-value |
| --- | --- | --- | --- | --- |
| Ln(West Single-family Starts(t-1)) | 0.96 | 0.03 | 34.89 | 0.00 |
| Q1 | 0.24 | 0.05 | 4.75 | 0.00 |
| Q2 | 0.48 | 0.03 | 17.58 | 0.00 |
| Q3 | 0.18 | 0.03 | 6.65 | 0.00 |
| D(Ln(US real GDP Per Capita)) | 5.18 | 1.85 | 2.81 | 0.01 |
| D(Ln(Mortgage Delinquency Rate)) | -0.10 | 0.18 | -0.55 | 0.58 |
| D(Ln(Mortgage Rate(t-1))) | -0.48 | 0.21 | -2.28 | 0.02 |
| D(Ln(U.S. Total Population)) | 22.16 | 20.70 | 1.07 | 0.29 |
| Constant | -0.13 | 0.11 | -1.19 | 0.24 |
| Number of Observations | 122 |  |  |  |
| F(8,113) | 241.83 |  |  |  |
| Prob > F | 0.00 |  |  |  |
| R^2^ | 0.96 |  |  |  |
| Root MSE | 0.10 |  |  |  |
| Durbin’s H-Statistic | -0.09 |  |  |  |
