# Supplementary material for: Housing starts and the associated wood products carbon storage by county by Shared Socioeconomic Pathway in the United States
Source: PLoS One. 2022 Aug 11;17(8):e0270025. doi: 10.1371/journal.pone.0270025 (PMC9371325; doi:10.1371/journal.pone.0270025)
Supplement: S9 Table — (DOCX) [file pone.0270025.s017.docx]

S9 Table. Northeast U.S. Census Region quarterly multifamily housing starts, least squares equation estimates; dependent variable natural log.

|  | Coefficient | Standard Error | t-value | p-value |
| --- | --- | --- | --- | --- |
| Ln(Northeast Multifamily Starts(t-1)) | 0.54 | 0.08 | 6.63 | 0.00 |
| Q1 |  |  |  |  |
| Q2 | 0.30 | 0.06 | 4.92 | 0.00 |
| Q3 | 0.37 | 0.06 | 6.10 | 0.00 |
| D(Ln(US real GDP)) | 11.90 | 8.53 | 1.40 | 0.17 |
| Ln(Northeast Multifamily Starts(t-2)) | 0.38 | 0.08 | 4.69 | 0.00 |
| Constant | -0.06 | 0.10 | -0.60 | 0.55 |
| Number of Observations | 121 |  |  |  |
| F(5,115) | 122.04 |  |  |  |
| Prob > F | 0.00 |  |  |  |
| R^2^ | 0.76 |  |  |  |
| Root MSE | 0.29 |  |  |  |
| Durbin’s H-Statistic | -0.46 |  |  |  |
