# Supplementary material for: Housing starts and the associated wood products carbon storage by county by Shared Socioeconomic Pathway in the United States
Source: PLoS One. 2022 Aug 11;17(8):e0270025. doi: 10.1371/journal.pone.0270025 (PMC9371325; doi:10.1371/journal.pone.0270025)
Supplement: S10 Table — (DOCX) [file pone.0270025.s018.docx]

S10 Table. Midwest U.S. Census Region quarterly multifamily housing starts, least squares equation estimates; dependent variable natural log.

|  | Coefficient | Standard Error | t-value | p-value |
| --- | --- | --- | --- | --- |
| Ln(Midwest Multifamily Starts(t-1)) | 0.33 | 0.05 | 6.54 | 0.00 |
| Q1 | -0.85 | 0.10 | -8.35 | 0.00 |
| Q2 |  |  |  |  |
| Q3 |  |  |  |  |
| D(Ln(US real GDP)) | 10.37 | 4.50 | 2.30 | 0.02 |
| D(Ln(Mortgage Delinquency Rate)) | -0.99 | 0.41 | -2.43 | 0.02 |
| Ln(Midwest Multifamily Starts(t-2)) | 0.22 | 0.05 | 4.17 | 0.00 |
| Ln(Midwest Multifamily Starts(t-3)) | 0.31 | 0.05 | 6.43 | 0.00 |
| Constant | 0.48 | 0.15 | 3.25 | 0.00 |
| Number of Observations | 120 |  |  |  |
| F(6,113) | 71.13 |  |  |  |
| Prob > F | 0.00 |  |  |  |
| R^2^ | 0.80 |  |  |  |
| Root MSE | 0.23 |  |  |  |
| Durbin’s H-Statistic | -0.10 |  |  |  |
