# Supplementary material for: Housing starts and the associated wood products carbon storage by county by Shared Socioeconomic Pathway in the United States
Source: PLoS One. 2022 Aug 11;17(8):e0270025. doi: 10.1371/journal.pone.0270025 (PMC9371325; doi:10.1371/journal.pone.0270025)
Supplement: S11 Table — (DOCX) [file pone.0270025.s019.docx]

S11 Table. South U.S. Census Region quarterly multifamily housing starts, least squares equation estimates; dependent variable natural log.

|  | Coefficient | Standard Error | t-value | p-value |
| --- | --- | --- | --- | --- |
| Ln(South Multifamily Starts(t-1)) | 0.87 | 0.06 | 15.36 | 0.00 |
| Q1 |  |  |  |  |
| Q2 | 0.11 | 0.04 | 2.56 | 0.01 |
| Q3 |  |  |  |  |
| D(Ln(US real GDP)) | 8.90 | 3.16 | 2.82 | 0.01 |
| Constant | 0.36 | 0.21 | 1.71 | 0.09 |
| Number of Observations | 122 |  |  |  |
| F(3,118) | 79.59 |  |  |  |
| Prob > F | 0.00 |  |  |  |
| R^2^ | 0.83 |  |  |  |
| Root MSE | 0.20 |  |  |  |
| Durbin’s H-Statistic | -0.41 |  |  |  |
