# Supplementary material for: Housing starts and the associated wood products carbon storage by county by Shared Socioeconomic Pathway in the United States
Source: PLoS One. 2022 Aug 11;17(8):e0270025. doi: 10.1371/journal.pone.0270025 (PMC9371325; doi:10.1371/journal.pone.0270025)
Supplement: S12 Table — (DOCX) [file pone.0270025.s020.docx]

S12 Table. West U.S. Census Region quarterly multifamily housing starts, least squares equation estimates; dependent variable natural log.

|  | Coefficient | Standard Error | t-value | p-value |
| --- | --- | --- | --- | --- |
| Ln(West Multifamily Starts(t-1)) | 0.64 | 0.13 | 4.77 | 0.00 |
| Q1 |  |  |  |  |
| Q2 | 0.33 | 0.06 | 5.83 | 0.00 |
| Q3 | 0.29 | 0.06 | 4.71 | 0.00 |
| D(Ln(US real GDP)) | 7.66 | 3.13 | 2.45 | 0.02 |
| D(Ln(Mortgage Delinquency Rate)) | -0.44 | 0.21 | -2.15 | 0.03 |
| Ln(West Multifamily Starts(t-2)) | 0.27 | 0.11 | 2.51 | 0.01 |
| Constant | 0.06 | 0.19 | 0.34 | 0.73 |
| Number of Observations | 121 |  |  |  |
| F(6,114) | 73.40 |  |  |  |
| Prob > F | 0.00 |  |  |  |
| R^2^ | 0.86 |  |  |  |
| Root MSE | 0.22 |  |  |  |
| Durbin’s H-Statistic | -0.47 |  |  |  |
