# Supplementary material for: Housing starts and the associated wood products carbon storage by county by Shared Socioeconomic Pathway in the United States
Source: PLoS One. 2022 Aug 11;17(8):e0270025. doi: 10.1371/journal.pone.0270025 (PMC9371325; doi:10.1371/journal.pone.0270025)
Supplement: S13 Table — (DOCX) [file pone.0270025.s021.docx]

S13 Table. Northeast U.S. Census Region quarterly single-family housing starts, Poisson pseudo-maximum likelihood equation estimates.

|  | Coefficient | Standard Error | t-value | p-value |
| --- | --- | --- | --- | --- |
| Northeast Single-family Starts(t-1) | 0.016 | 0.003 | 6.15 | 0 |
| Q1 | -0.35 | 0.11 | -3.17 | 0.00 |
| Q2 | 0.22 | 0.10 | 2.16 | 0.03 |
| Q3 | 0.16 | 0.08 | 1.86 | 0.06 |
| D(Ln(US real GDP Per Capita)) | 7.17 | 2.90 | 2.47 | 0.01 |
| D(Mortgage Delinquency Rate) | -0.041 | 0.062 | -0.67 | 0.50 |
| D(Mortgage Rate(t-1)) | -0.032 | 0.032 | -1.00 | 0.32 |
| D(Ln(Northeast Population)) | 104.57 | 46.54 | 2.25 | 0.03 |
| Northeast Single-family Starts(t-2) | 0.0066 | 0.0037 | 1.81 | 0.07 |
| Northeast Single-family Starts(t-3) | 0.0075 | 0.0043 | 1.75 | 0.08 |
| Northeast Single-family Starts(t-4) | 0.0067 | 0.0043 | 1.57 | 0.12 |
| Northeast Single-family Starts(t-5) | -0.0073 | 0.0039 | -1.87 | 0.06 |
| Constant | 2.26 | 0.09 | 26.32 | 0.00 |
| Number of Observations | 118 |  |  |  |
| Wald χ^2^ (12) | 1279.25 |  |  |  |
| Prob > χ^2^ | 0.00 |  |  |  |
| Pseudo R^2^ | 0.48 |  |  |  |
