# Supplementary material for: Housing starts and the associated wood products carbon storage by county by Shared Socioeconomic Pathway in the United States
Source: PLoS One. 2022 Aug 11;17(8):e0270025. doi: 10.1371/journal.pone.0270025 (PMC9371325; doi:10.1371/journal.pone.0270025)
Supplement: S14 Table — (DOCX) [file pone.0270025.s022.docx]

S14 Table. Midwest U.S. Census Region quarterly single-family housing starts, Poisson pseudo-maximum likelihood equation estimates.

|  | Coefficient | Standard Error | t-value | p-value |
| --- | --- | --- | --- | --- |
| Midwest Single-family Starts(t-1) | 0.013 | 0.002 | 7.66 | 0 |
| Q1 | -0.41 | 0.11 | -3.83 | 0.00 |
| Q2 | 0.41 | 0.10 | 4.22 | 0.00 |
| Q3 | 0.31 | 0.10 | 3.06 | 0.00 |
| D(Ln(US real GDP Per Capita)) | 5.89 | 1.76 | 3.35 | 0.00 |
| D(Mortgage Delinquency Rate) | -0.12 | 0.05 | -2.59 | 0.01 |
| D(Mortgage Rate(t-1)) | -0.11 | 0.03 | -3.61 | 0.00 |
| D(Ln(Midwest Population)) | 97.89 | 17.23 | 5.68 | 0.00 |
| Midwest Single-family Starts(t-2) | 0.0063 | 0.0018 | 3.51 | 0.00 |
| Midwest Single-family Starts(t-3) | 0.0007 | 0.0020 | 0.34 | 0.74 |
| Midwest Single-family Starts(t-4) | 0.0037 | 0.0019 | 1.88 | 0.06 |
| Midwest Single-family Starts(t-5) | -0.0058 | 0.0021 | -2.76 | 0.01 |
| Midwest Single-family Starts(t-6) | -0.0007 | 0.0018 | -0.37 | 0.71 |
| Midwest Single-family Starts(t-7) | 0.0043 | 0.0015 | 2.77 | 0.01 |
| Constant | 2.49 | 0.07 | 34.42 | 0.00 |
| Number of Observations | 116 |  |  |  |
| Wald χ^2^ (14) | 2736.48 |  |  |  |
| Prob > χ^2^ | 0.00 |  |  |  |
| Pseudo R^2^ | 0.62 |  |  |  |
