# Supplementary material for: Housing starts and the associated wood products carbon storage by county by Shared Socioeconomic Pathway in the United States
Source: PLoS One. 2022 Aug 11;17(8):e0270025. doi: 10.1371/journal.pone.0270025 (PMC9371325; doi:10.1371/journal.pone.0270025)
Supplement: S15 Table — (DOCX) [file pone.0270025.s023.docx]

S15 Table. South U.S. Census Region quarterly single-family housing starts, Poisson pseudo-maximum likelihood equation estimates.

|  | Coefficient | Standard Error | t-value | p-value |
| --- | --- | --- | --- | --- |
| South Single-family Starts(t-1) | 0.0075 | 0.0003 | 25.2 | 0 |
| Q1 | 0.15 | 0.03 | 4.53 | 0.00 |
| Q2 | 0.33 | 0.02 | 14.42 | 0.00 |
| Q3 | 0.11 | 0.02 | 4.98 | 0.00 |
| D(Ln(US real GDP Per Capita)) | 7.40 | 1.43 | 5.16 | 0.00 |
| D(Mortgage Delinquency Rate) | -0.075 | 0.027 | -2.77 | 0.01 |
| D(Mortgage Rate(t-1)) | -0.072 | 0.021 | -3.46 | 0.00 |
| D(Ln(South Population)) | 53.67 | 12.70 | 4.23 | 0.00 |
| Constant | 3.47 | 0.06 | 56.46 | 0.00 |
| Number of Observations | 122 |  |  |  |
| Wald χ^2^ (8) | 1241.29 |  |  |  |
| Prob > χ^2^ | 0.00 |  |  |  |
| Pseudo R^2^ | 0.64 |  |  |  |
