# Supplementary material for: Housing starts and the associated wood products carbon storage by county by Shared Socioeconomic Pathway in the United States
Source: PLoS One. 2022 Aug 11;17(8):e0270025. doi: 10.1371/journal.pone.0270025 (PMC9371325; doi:10.1371/journal.pone.0270025)
Supplement: S16 Table — (DOCX) [file pone.0270025.s024.docx]

S16 Table. West U.S. Census Region quarterly single-family housing starts, Poisson pseudo-maximum likelihood equation estimates.

|  | Coefficient | Standard Error | t-value | p-value |
| --- | --- | --- | --- | --- |
| West Single-family Starts(t-1) | 0.0160 | 0.0005 | 32.35 | 0 |
| Q1 | 0.27 | 0.05 | 5.07 | 0.00 |
| Q2 | 0.47 | 0.03 | 15.93 | 0.00 |
| Q3 | 0.14 | 0.03 | 4.40 | 0.00 |
| D(Ln(US real GDP Per Capita)) | 4.33 | 2.01 | 2.15 | 0.03 |
| D(Mortgage Delinquency Rate) | 0.020 | 0.039 | 0.51 | 0.61 |
| D(Mortgage Rate(t-1)) | -0.056 | 0.028 | -2.02 | 0.04 |
| D(Ln(West Population)) | 79.32 | 11.30 | 7.02 | 0.00 |
| Constant | 2.50 | 0.07 | 36.49 | 0.00 |
| Number of Observations | 122 |  |  |  |
| Wald χ^2^ (8) | 1556.78 |  |  |  |
| Prob > χ^2^ | 0.00 |  |  |  |
| Pseudo R^2^ | 0.59 |  |  |  |
