# Supplementary material for: Housing starts and the associated wood products carbon storage by county by Shared Socioeconomic Pathway in the United States
Source: PLoS One. 2022 Aug 11;17(8):e0270025. doi: 10.1371/journal.pone.0270025 (PMC9371325; doi:10.1371/journal.pone.0270025)
Supplement: S17 Table — (DOCX) [file pone.0270025.s025.docx]

S17 Table. Northeast U.S. Census Region quarterly multifamily housing starts, Poisson pseudo-maximum likelihood equation estimates.

|  | Coefficient | Standard Error | t-value | p-value |
| --- | --- | --- | --- | --- |
| Northeast Multifamily Starts(t-1) | 0.055 | 0.006 | 8.68 | 0 |
| Q1 | -0.32 | 0.14 | -2.35 | 0.02 |
| Q2 | 0.31 | 0.09 | 3.58 | 0.00 |
| Q3 | 0.40 | 0.07 | 5.86 | 0.00 |
| D(Ln(US real GDP)) | 6.81 | 6.58 | 1.04 | 0.30 |
| D(Mortgage Delinquency Rate) | -0.23 | 0.13 | -1.83 | 0.07 |
| D(Mortgage Rate(t-1)) | -0.069 | 0.067 | -1.03 | 0.30 |
| Northeast Multifamily Starts(t-2) | 0.037 | 0.008 | 4.37 | 0.00 |
| Constant | 1.14 | 0.09 | 12.07 | 0.00 |
| Number of Observations | 121 |  |  |  |
| Wald χ^2^ (8) | 395.51 |  |  |  |
| Prob > χ^2^ | 0.00 |  |  |  |
| Pseudo R^2^ | 0.27 |  |  |  |
