# Supplementary material for: Housing starts and the associated wood products carbon storage by county by Shared Socioeconomic Pathway in the United States
Source: PLoS One. 2022 Aug 11;17(8):e0270025. doi: 10.1371/journal.pone.0270025 (PMC9371325; doi:10.1371/journal.pone.0270025)
Supplement: S18 Table — (DOCX) [file pone.0270025.s026.docx]

S18 Table. Midwest U.S. Census Region quarterly multifamily housing starts, Poisson pseudo-maximum likelihood equation estimates.

|  | Coefficient | Standard Error | t-value | p-value |
| --- | --- | --- | --- | --- |
| Midwest Multifamily Starts(t-1) | 0.024 | 0.003 | 7.83 | 0 |
| Q1 | -0.85 | 0.10 | -8.94 | 0.00 |
| Q2 |  |  |  |  |
| Q3 |  |  |  |  |
| D(Ln(US real GDP)) | 10.89 | 3.42 | 3.18 | 0.00 |
| D(Mortgage Delinquency Rate) | -0.21 | 0.08 | -2.73 | 0.01 |
| D(Mortgage Rate(t-1)) | -0.083 | 0.053 | -1.58 | 0.12 |
| Midwest Multifamily Starts(t-2) | 0.012 | 0.003 | 3.54 | 0.00 |
| Midwest Multifamily Starts(t-3) | 0.020 | 0.004 | 5.70 | 0.00 |
| Constant | 1.91 | 0.08 | 25.22 | 0.00 |
| Number of Observations | 120 |  |  |  |
| Wald χ^2^ (7) | 447.66 |  |  |  |
| Prob > χ^2^ | 0.00 |  |  |  |
| Pseudo R^2^ | 0.31 |  |  |  |
