# Supplementary material for: Housing starts and the associated wood products carbon storage by county by Shared Socioeconomic Pathway in the United States
Source: PLoS One. 2022 Aug 11;17(8):e0270025. doi: 10.1371/journal.pone.0270025 (PMC9371325; doi:10.1371/journal.pone.0270025)
Supplement: S19 Table — (DOCX) [file pone.0270025.s027.docx]

S19 Table. South U.S. Census Region quarterly multifamily housing starts, Poisson pseudo-maximum likelihood equation estimates.

|  | Coefficient | Standard Error | t-value | p-value |
| --- | --- | --- | --- | --- |
| South Multifamily Starts(t-1) | 0.019 | 0.002 | 11.56 | 0 |
| Q1 |  |  |  |  |
| Q2 | 0.14 | 0.04 | 3.53 | 0.00 |
| Q3 |  |  |  |  |
| D(Ln(US real GDP)) | 9.35 | 3.17 | 2.95 | 0.00 |
| D(Mortgage Delinquency Rate) | -0.016 | 0.035 | -0.44 | 0.66 |
| D(Mortgage Rate(t-1)) | -0.039 | 0.052 | -0.75 | 0.45 |
| Constant | 2.70 | 0.06 | 44.50 | 0.00 |
| Number of Observations | 122 |  |  |  |
| Wald χ^2^ (5) | 295.89 |  |  |  |
| Prob > χ^2^ | 0.00 |  |  |  |
| Pseudo R^2^ | 0.42 |  |  |  |
