# Supplementary material for: Housing starts and the associated wood products carbon storage by county by Shared Socioeconomic Pathway in the United States
Source: PLoS One. 2022 Aug 11;17(8):e0270025. doi: 10.1371/journal.pone.0270025 (PMC9371325; doi:10.1371/journal.pone.0270025)
Supplement: S20 Table — (DOCX) [file pone.0270025.s028.docx]

S20 Table. West U.S. Census Region quarterly multifamily housing starts, Poisson pseudo-maximum likelihood equation estimates.

|  | Coefficient | Standard Error | t-value | p-value |
| --- | --- | --- | --- | --- |
| West Multifamily Starts(t-1) | 0.016 | 0.004 | 3.93 | 0 |
| Q1 |  |  |  |  |
| Q2 | 0.33 | 0.06 | 5.70 | 0.00 |
| Q3 | 0.29 | 0.05 | 5.50 | 0.00 |
| D(Ln(US real GDP)) | 8.43 | 3.07 | 2.74 | 0.01 |
| D(Mortgage Delinquency Rate) | -0.088 | 0.046 | -1.92 | 0.06 |
| D(Mortgage Rate(t-1)) | 0.000 | 0.048 | 0.00 | 1.00 |
| West Multifamily Starts(t-2) | 0.015 | 0.004 | 4.13 | 0.00 |
| Constant | 2.11 | 0.06 | 36.83 | 0.00 |
| Number of Observations | 121 |  |  |  |
| Wald χ^2^ (7) | 530.58 |  |  |  |
| Prob > χ^2^ | 0.00 |  |  |  |
| Pseudo R^2^ | 0.47 |  |  |  |
