# Supplementary material for: Housing starts and the associated wood products carbon storage by county by Shared Socioeconomic Pathway in the United States
Source: PLoS One. 2022 Aug 11;17(8):e0270025. doi: 10.1371/journal.pone.0270025 (PMC9371325; doi:10.1371/journal.pone.0270025)
Supplement: S21 Table — (DOCX) [file pone.0270025.s029.docx]

S21 Table. Least squares regression of the first-difference in the natural logarithm of real U.S. GDP, quarterly, 1984Q1-2014Q3.

|  | Coefficient | Standard Error | t-value | p-value |
| --- | --- | --- | --- | --- |
| D(Ln(Real GDP*_t_*_-1_)) | 0.43 | 0.10 | 4.18 | 0.00 |
| D(Ln(Real GDP*_t_*_-8_)) | -0.14 | 0.08 | -1.76 | 0.08 |
| D(Ln(Real GDP*_t_*_-9_)) | 0.25 | 0.09 | 2.91 | 0.00 |
| D(Ln(Real GDP*_t_*_-12_)) | -0.14 | 0.06 | -2.32 | 0.02 |
| Q2 dummy | 0.0044 | 0.0013 | 3.37 | 0.00 |
| Constant | 0.0029 | 0.0012 | 2.33 | 0.02 |
| Number of Observations | 122 |  |  |  |
| F( 5, 116) | 4.81 |  |  |  |
| Prob > F | 0.0005 |  |  |  |
| R-squared | 0.26 |  |  |  |
| Root MSE | 0.0054 |  |  |  |
| Durbin’s H-Statistic | -0.42 |  |  |  |
