# Supplementary material for: Housing starts and the associated wood products carbon storage by county by Shared Socioeconomic Pathway in the United States
Source: PLoS One. 2022 Aug 11;17(8):e0270025. doi: 10.1371/journal.pone.0270025 (PMC9371325; doi:10.1371/journal.pone.0270025)
Supplement: S22 Table — (DOCX) [file pone.0270025.s030.docx]

S22 Table. Least squares regression of the natural logarithm of the total mortgage delinquency rate in the United States, quarterly, 1984Q1-2014Q3.

|  | Coefficient | Standard Error | t-value | p-value |
| --- | --- | --- | --- | --- |
| Ln(Mortgage Delinquency Rate*_t_*_-1_) | 0.94 | 0.02 | 54.14 | 0.00 |
| D(Ln(Mortgage Delinquency Rate*_t_*_-4_)) | 0.39 | 0.08 | 4.92 | 0.00 |
| D(Ln(US real GDP*_t_*_-1_)) | -2.75 | 0.83 | -3.33 | 0.00 |
| Q1 dummy | -0.12 | 0.02 | -6.38 | 0.00 |
| Q2 dummy | -0.019 | 0.009 | -1.97 | 0.05 |
| Constant | 0.16 | 0.03 | 4.90 | 0.00 |
| Number of Observations | 122 |  |  |  |
| F( 5, 116) | 872.56 |  |  |  |
| Prob > F | 0 |  |  |  |
| R-squared | 0.97 |  |  |  |
| Root MSE | 0.046 |  |  |  |
| Durbin’s H-Statistic | -0.07 |  |  |  |
