# Supplementary material for: Housing starts and the associated wood products carbon storage by county by Shared Socioeconomic Pathway in the United States
Source: PLoS One. 2022 Aug 11;17(8):e0270025. doi: 10.1371/journal.pone.0270025 (PMC9371325; doi:10.1371/journal.pone.0270025)
Supplement: S23 Table — (DOCX) [file pone.0270025.s031.docx]

S23 Table. Least squares regression of the first-difference in the natural logarithm of the nominal mortgage interest rate, quarterly, 1984Q1-2014Q3.

|  | Coefficient | Standard Error | t-value | p-value |
| --- | --- | --- | --- | --- |
| Q2 dummy | 0.025 | 0.010 | 2.37 | 0.019 |
| D(Ln(Real GDP*_t_*)) | 2.50 | 0.71 | 3.54 | 0.001 |
| Constant | -0.033 | 0.007 | -4.87 | 0.000 |
| Number of Observations | 122 |  |  |  |
| F( 5, 116) | 9.70 |  |  |  |
| Prob > F | 0.0001 |  |  |  |
| R-squared | 0.14 |  |  |  |
| Root MSE | 0.049 |  |  |  |
| Durbin’s H-Statistic | 0.09 |  |  |  |
